# Invasion triangle: an organizational framework for species invasion

**DOI:** 10.1002/ece3.47

**Published:** 2011-12

**Authors:** Lora B Perkins, Elizabeth A Leger, Robert S Nowak

**Affiliations:** Department of Natural Resources and Environmental Science, University of NevadaReno, Nevada

**Keywords:** Biological invasion, Conceptual framework, Invasive species, Nonnative, Species introduction

## Abstract

Species invasion is a complex, multifactor process. To encapsulate this complexity into an intuitively appealing, simple, and straightforward manner, we present an organizational framework in the form of an invasion triangle. The invasion triangle is an adaptation of the disease triangle used by plant pathologists to help envision and evaluate interactions among a host, a pathogen, and an environment. Our modification of this framework for invasive species incorporates the major processes that result in invasion as the three sides of the triangle: (1) attributes of the potential invader; (2) biotic characteristics of a potentially invaded site; and (3) environmental conditions of the site. The invasion triangle also includes the impact of external influences on each side of the triangle, such as climate and land use change. This paper introduces the invasion triangle, discusses how accepted invasion hypotheses are integrated in this framework, describes how the invasion triangle can be used to focus research and management, and provides examples of application. The framework provided by the invasion triangle is easy to use by both researchers and managers and also applicable at any level of data intensity, from expert opinion to highly controlled experiments. The organizational framework provided by the invasion triangle is beneficial for understanding and predicting why species are invasive in specific environments, for identifying knowledge gaps, for facilitating communication, and for directing management in regard to invasive species.

## Introduction

The geographic barriers that maintain species in their native ranges are being overcome at an increasing rate, resulting in a global homogenization of species (Mack et al. 2000; Harris et al. 2011). Species migrations that formerly took place over geologic time scales now occur in the time scale it takes for a cross-oceanic flight. Although only a fraction of introduced species become invasive (Richardson et al. 2000b), any increase in the number of introductions will increase probability of invasion. Furthermore, a growing consensus acknowledges that invasion is not a result of a single factor; most invasions occur due to a combination of factors (Lonsdale 1999; Alpert et al. 2000; Davis et al. 2000; Richardson et al. 2000b; Facon et al. 2006; Barney and Whitlow 2008; Catford et al. 2009; Foxcroft et al. 2011). Nonetheless, despite many years of study, many proposed hypotheses, and many theories about the factors that contribute to invasion, invasion ecology still has no widely accepted, unified paradigm to evaluate the risk of invasion (Richardson et al. 2000b; Barney and Whitlow 2008; Catford et al. 2009; Gurevitch et al. 2011).

**Figure 1:**
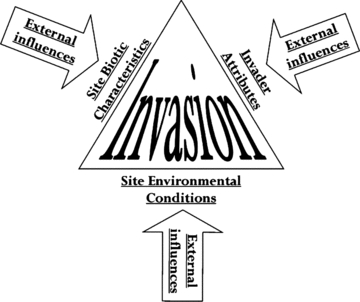
Conceptual diagram of the invasion triangle. Invader attributes refer to inherent characteristics of the introduced species (including competitive ability, novel weapons, evolution of invasiveness, and ecosystem engineering). Site biotic characteristics refer to the intrinsic biological characteristics of a site that influence its vulnerability to invasion (including diversity and the presence of potential enemies and mutualists). Site environmental conditions refer to the environmental or physical conditions of a site that influence its invasibility (including the amount of unused resources and habitat suitability). Invader attributes, site biotic characteristics, and site environmental conditions all interact to determine the potential for invasion. The external influences arrows indicate that all sides can be changed by outside factors, such as introduction effort, global environmental change, or changes in land use and disturbance regime.

In this paper, we describe an organizational framework in the form of an invasion triangle that embodies the multifactor process of invasion (Fig. 1). At the core of the invasion triangle are three categories of factors that influence invasion: (1) attributes of the potential invader; (2) biotic characteristics of a potentially invaded site; and (3) environmental conditions of a potentially invaded site. The invasion triangle also incorporates external influences such as climate change (Hellmann et al. 2008; Walther et al. 2009), land use change (Hobbs and Huenneke 1992; Jesson et al. 2000), and external inputs such as nitrogen deposition (Fenn et al. 2003) that affect invasion either directly or by influencing the three categories mentioned above (Fig. 1).

The invasion triangle is a conceptual framework that is adaptable to any potential invasive species and any potentially invaded site. This adaptability allows for the invasion triangle to provide connection and consistency among the very diverse projects that examine invasion. Consistency provides a “step back to get a larger view” (Davis et al. 2005) and facilitates identification of larger patterns in invasion. Increased understanding of invasion as a whole and identification of larger patterns requires consistent consideration of many influential factors in multiple invasion situations. The invasion triangle is a conceptual framework that explicitly requires consideration of many influential factors, is adaptable to any invasion situation, and can therefore provide the consistency among projects needed for greater understanding of the causes of invasion.

In this paper, we introduce the invasion triangle, describe how it can be used, provide examples of invasion triangle application, and briefly discuss next steps that can move it from a conceptual framework into a quantitative model. To introduce the invasion triangle, we summarize factors that correspond to each side of the triangle and discuss external influences. We organize our summary of factors around a nonexhaustive subset of hypotheses that have been proposed to explain invasion.

### Definitions

In this paper, we use the term “invasion” to refer to the rapid spread and increase in dominance of a population of nonnative species in a recipient ecosystem (sensu Valery et al. 2008). An “invasive species” is defined as a nonnative species that spreads and produces negative effects on the resident community (Alpert et al. 2000). We consider “site invasibility” to mean the relative susceptibility of a site to invasion and define “species invasive potential” as the ability of a species to invade in a particular environment (Williamson 1996).

### Background

The invasion triangle is an adaptation of the disease triangle used by plant pathologists to help envision and evaluate the interactions among a host, a pathogen, and an environment. These interactions are depicted as sides of the disease triangle and determine the extent of disease development. This plant pathology disease triangle has a long history of use (Stevens 1960) and is considered a central principle of plant pathology (Parker and Gilbert 2004). By combining all three aspects of plant disease into a single triangular structure, the disease triangle is based on the clear understanding that a combination of all three factors is necessary for disease to develop. For example, even if a susceptible host is exposed to a virulent pathogen, disease will not develop without a conducive environment, or similarly, a susceptible host in a conducive environment will not develop disease if a virulent pathogen is not present (Parker and Gilbert 2004). Here, we adapt the plant disease triangle into an invasion triangle, in which we substitute the invader for the pathogen, the biotic characteristics of the potentially invaded site for the host, modify the environmental variables, and introduce external influences to all three sides of the triangle. A defining characteristic of the invasion triangle is the graphical representation of all the factors involved in invasion. Examining any one side of the triangle individually is worthwhile and beneficial, but to evaluate invasion risk and management of a particular introduction at a particular location and to contribute to a larger understanding of invasion as a whole, factors on all three sides and the effects of external influences must be considered. To fully understand how one aspect of invasion (e.g., the biotic characteristics or environmental conditions of a site or invader attributes) affects invasion success, other aspects must also be considered (Lonsdale 1999).

Other comprehensive frameworks have certainly been proposed for invasion (Lonsdale 1999; Richardson et al. 2000b; Barney and Whitlow 2008; Catford et al. 2009; Foxcroft et al. 2011). The invasion triangle builds and improves upon these other frameworks. A major difference between other frameworks and the invasion triangle is the deliberate separation of the sets of factors depicted on each side (site biotic characteristics, site environmental conditions, and invader attributes) and external influences within a single cohesive framework. This separation allows for (1) independent evaluation of the factors affecting each side of the triangle; (2) consideration of the interactions between sides (i.e., the importance of different invasive attributes for invasion into sites with different site environmental conditions and with different site biotic characteristics); and (3) impacts of external influences to be evaluated independently on each side. The factors depicted on different sides of the invasion triangle cannot be assumed to respond similarly to external influences. For example, site biotic characteristics may not respond to global environmental changes in the same direction or magnitude as potential invaders respond, which may be differentially susceptible to change than species present in a given environment. External influences (e.g., alteration of land use or global environmental change) must also be incorporated into any conceptual framework of invasion and should be considered as a separate set of factors that can potentially influence other processes independently. Another minor difference between previous frameworks and the invasion triangle is the invasion triangle is applicable to all invasive species, not just invasive plants. Although the invasion triangle and the previous frameworks share many (but not all) of the same factors, the structure of the invasion triangle makes it the most effective and usable framework.

## Structure of the invasion triangle

A key element of the invasion triangle is its three sides: invader attributes, site biotic characteristics, and site environmental conditions (Fig. 1). Each is introduced briefly here and developed in greater detail below. Invader attributes are intrinsic characteristics of a species that influence its ability to become invasive. The invasive potential of a species increases with the number of invasive attributes it displays or through stronger manifestation of particular invasive attributes. Site biotic characteristics are the intrinsic biological characteristics of a recipient site that relate to its vulnerability to invasion. The risk of invasion increases as the resistance to invasion provided by the biotic characteristics present at a site decreases. Site environmental conditions are the inherent physical conditions of a recipient site that influence invisibility to a particular species. Invasion becomes more likely as the environmental conditions at a site become more conducive to the invader's success.

An underlying assumption of the invasion triangle is that the size of the triangle is directly proportional to invasion. Intuitively, as each side of the triangle (i.e., invader attributes, site biotic characteristics, and site environmental characteristics) increases for a particular invasion into a specific area, that species should be more successful invading that area. Increased length of one or more sides increases overall triangle size. Thus, triangle size and probability of invasion should be proportional. This assumption and the methods needed to quantify the size of invasion triangles are discussed in more detail in the “Next Steps” section near the end of this paper.

**Figure 2:**
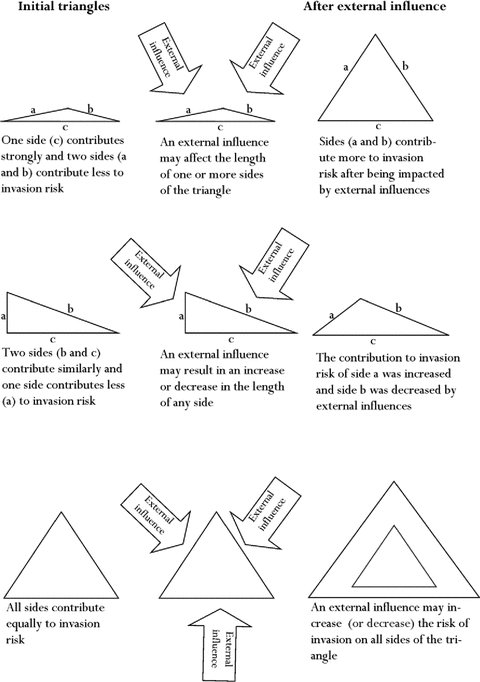
Illustration of how scaled invasion triangles communicate information on invasion. If sides are scaled relative to each other (see Next Steps section for further discussion on the quantitative aspects of the invasion triangle), then longer sides will indicate more influence on invasion than shorter sides. External influences may affect one or more “sides” of the invasion triangle.

Other key elements of the invasion triangle are incorporation of external influences, that is, transient dynamics generated outside of a site and the implicit interaction between sides. External influences (e.g., wildfire or livestock grazing) modify the invasibility of a site or change the invasive potential of a species. An external influence can act on one or more sides of the invasion triangle (Fig. 2), potentially impacting one or more factors impacting invasion. In any invasion situation, the factors depicted on each side of the triangle may interact. For example, site biotic characteristics and site environmental conditions are closely linked and changes in one may cause changes in the other. The interactions among factors depicted on each side should be considered on a site- and species-specific basis if needed.

### Invader attributes

The first set of factors discussed is depicted as the “Invader Attributes” side of the invasion triangle and represents the intrinsic characteristics of a species that affect its invasive potential. More or stronger attributes increase the invasive potential of a species. Much of the early research on invasions focused on defining characteristics common to invasive species, with the idea that a core suite of life-history traits (i.e., rapid growth rate, short generation time, or high reproductive capacity) might be identified that strongly predisposes a species to becoming invasive (Baker 1974). Clearly, the ability to predict which species have more invasive potential would be a very powerful tool for conservation and management. However, life-history traits that are consistently associated with invasive potential have, so far, proven difficult to determine (Alpert et al. 2000; Sakai et al. 2001). Because traits that confer an invasive advantage may differ among the wide range of ecosystems experiencing invasion, identification of a single “invasive” life-history strategy may be futile. Nevertheless, invasion ecology has produced many hypotheses that relate to strategies, or suites of traits, that may influence species invasive potential. Specific hypotheses that are discussed in relation to the invasion triangle are: competitive ability, novel weapons, evolution of invasiveness, and engineering.

#### Competitive ability

The hypothesis that invasive species are better competitors than native species is long standing with documented support in many ecosystems (Elton 1958; Sakai et al. 2001; Vila and Wiener 2004). This hypothesis broadly states that invaders are inherently superior competitors compared with native species. Competition is defined as both direct and indirect effects of invader on neighbors (Vila and Weiner 2004). An invader with the ability to produce a large competitive effect (i.e., ability to suppress neighbors) and a robust competitive response (i.e., avoidance of suppression due to neighbors; Goldberg and Landa 1991) will have greater invasive potential than species without those abilities. The competition hypothesis is often tested under experimental conditions and provides useful insight into the invasion potential of species.

#### Novel weapons

The novel weapons hypothesis proposes that an introduced species may possess traits that do not exist in resident species of a particular community and thus bring new mechanisms of interaction to which members of the recipient community are susceptible (Callaway and Aschehoug 2000). These new mechanisms decrease native species performance more than would be predicted by considering competitive relationships alone. The novel weapons hypothesis contends that the invasive species has a “weapon,” or nontrophic, noncompetitive mechanism of interaction (e.g., allelochemical deposition), to which members of its native community are conditioned and hence give the weapon negligible effect. In contrast, in the recipient site, the community is naïve or not conditioned to the weapon, and thus the weapon has a significant negative effect on members of the recipient site (Hierro et al. 2005). To evaluate this hypothesis, knowledge of effects of the invader on both naïve and experienced neighbors should be evaluated (Hierro et al. 2005). Members of the new recipient community must show much more susceptibility to the weapons of the potential invader than members of its home community. Species with novel weapons are expected to have greater invasive potential than species without.

An example of a species demonstrating a novel weapon is the glassy-winged sharpshooter *Homalodisca coagulata* in the invaded range of Tahiti and Mo'orea (Suttle and Hoddle 2006). Generalist predators are lethally intoxicated after preying upon *H. coagulata* in the invaded range (Suttle and Hoddle 2006). Generalist predators in the home range of *H. coagulata* are not similarly impacted (Suttle and Hoddle 2006). This weapon can be considered novel due to the contrasting reactions of generalist predators in the native and invaded range. This novel weapon contributes to the invasive potential of *H. coagulata* (Suttle and Hoddle 2006).

#### Evolution of invasiveness

Some seemingly benign species increase invasive potential after arrival on a recipient site. Genetic drift, high genetic diversity in the founder population, or a change in selection pressures can interact to produce invader populations that have greater invasive potential than populations in the home range (Blossey and Notzold 1995, Lambrinos 2004; Bossdorf et al. 2005; Schierenbeck and Ellstrand 2009; Buswell et al. 2011; Clements and Ditommaso 2011). An introduced species also has the capability to increase its invasive potential through hybridization with native species (Bossdorf et al. 2005). This evolution of invasiveness is occasionally linked to the concept of “enemy release”: when introduced into a new site, a species experiences little pressure from enemies (i.e., herbivores or pathogens), and thus less energy needs to be allocated to defense, that in turn gives an evolutionary advantage to individual plants that invest more energy in growth and reproduction (Blossey and Notzold 1995). Like many other hypotheses, support for evolution of invasiveness is contradictory, with this process occurring in some invasions but not others, possibly due to the many factors interacting in invasion. This hypothesis also may seem ex post facto, but it points toward the importance of using introduced populations for research regarding invasive potential and the careful monitoring of introduced populations with unknown invasive potential.

#### Engineering

An emerging invasion hypothesis relates to how species alter their environment through nontrophic interactions, or “ecosystem engineer” to promote themselves and decrease neighbors (Jones et al. 1994; Cuddington and Hastings 2004; Radford et al. 2010). These changes in environment can either be created actively (i.e., nitrogen fixation by invasive plants, Haubensak et al. 2011) or passively through growth (i.e., invasive seagrasses alter sedimentation rates, Cuddington and Hasting 2004). Engineering effects range dramatically in size from very large (tree canopies altering relative humidity and temperature, Jones et al. 1994) to very small (rhizosphere changes in pH; Radford et al. 2010). The impact of the engineering is possibly more important than the physical size of the impacted area, and ecosystem engineering status may only matter if a species alters the environment enough to affect population dynamics (Cuddington et al. 2009). A species that has the ability to alter population dynamics at a potentially invaded site by altering the physical environment (ecosystem engineering) has a greater invasive potential compared to species without that ability.

An example of an invader with high engineering ability is the hybrid cattail *Typha* X *glauca* in Great Lakes coastal marshes (Farrer and Goldberg 2009). Through abundant production of litter, *T.* X *glauca* engineers increased soil N, promotes its own growth, and suppresses native species (Farrer and Goldberg 2009). The considerable ability of *T.* X *glauca* to engineer its ecosystem certainly contributes to its invasive potential.

### Site Biotic Characteristics

The second set of factors discussed is depicted as the “Site Biotic Characteristics” side of the invasion triangle and represents site invasibility due to intrinsic biological characteristics of a site that influence its vulnerability to invasion. Here, we define biotic resistance as the cumulative ability of all the resident species on a site to resist invasion (Elton 1958; Sakai et al. 2001; Kennedy et al. 2002). Mechanistically, three different biotic characteristics of the recipient community independently influence the invasibility of a site and confer biotic resistance. These characteristics are biodiversity, enemies, and mutualists (Richardson et al. 2000a; Mitchell et al. 2006). We discuss each characteristic and its associated hypothesis individually.

#### Diversity

Another of the oldest hypotheses regarding invasion is the diversity hypothesis, which proposes that sites with more diverse communities are less susceptible to invasion (Elton 1958). That is, sites with higher numbers of species, higher diversity of species, or higher functional diversity of species are more resistant to invasion than species-impoverished sites. Sites with more diversity may have increased resistance to invasion in two ways: (1) as diversity increases, species more completely utilize resources, and thus leave less unused resources for a potential invader; and (2) more diverse communities have a higher probability that a species already present in the recipient community has the ability to successfully compete with or otherwise resist an invader (Wardle 2001). This hypothesis has been hotly debated with both supportive and contradictory evidence (Levine 2000; Mack et al. 2000; Wardle 2001; Kennedy et al. 2002; Stohlgren et al. 2003). This contradictory evidence suggests that diversity is only one of the many components in potential invasibility of a site, not the definitive barrier to invasion (Levine et al. 2004), and that differences in scale across which diversity is examined also influence the importance of diversity to invasibility (Shea and Chesson 2002).

#### Enemies

Two contrasting hypotheses consider the effects of enemies on site invasibility, depending on whether potential enemies of the invader are found in the recipient site. Natural enemies include pathogens, predators, or herbivores; essentially any member of the community that negatively affects growth, reproduction, or survival through a mechanism that is not competition.

The first enemy-related hypothesis, broadly referred to as the biotic resistance hypothesis (Maron and Vila 2001), considers the case where enemies of the introduced species are found in the recipient site but not found in the introduced species’ native area. This hypothesis proposes that enemies indigenous to a recipient site may limit the growth of a potentially invasive species, and consequently increases resistance to invasion and decreases site invasibility (Maron and Vila 2001). Thus, the presence of species that act as natural enemies to a given invader increases the resistance of a site to invasion by that invader.

The second enemy-related hypothesis considers the case where enemies that are typically found in the invader's native area are not present in the new, recipient site (i.e., the enemy release hypothesis, Keane and Crawley 2002; or natural enemy escape opportunity, Shea and Chesson 2002). The enemy release hypothesis suggests that as species are removed from their native range, they are removed from natural enemies that limit their growth in the introduced range. If the recipient site has no analogous enemies, then invasive species in the introduced range are free to grow optimally without limiting interactions with herbivores and pathogens (Keane and Crawley 2002). Although the presence of one effective enemy may be enough to provide resistance to invasion, a site depauperate in species that may potentially act as enemies to invaders generally would be more invasible than sites with many potential enemies.

#### Mutualists

Many invasions are promoted by the establishment of facilitative relationships formed between an invasive species and other species in the recipient community (Richardson et al. 2000a). Although technically these facilitative relationships can be either mutualistic (+/+) or commensalistic (+/0), for ease of communication, we use a less stringent definition of mutualism that includes both interaction types. A recipient community rich in potential mutualists has less biotic resistance and is more invasible than a community depauperate in mutualists (Shea and Chesson 2002). An invader may form positive relationships with the same species as in the home range or, because of the diffuse nature of most mutualisms, with novel species in the recipient community (Richardson et al. 2000a). The presence of other invasive species also has the potential to increase the likelihood of invasion (invasional meltdown; Simberloff and Von Holle 1999). Invasional meltdown is the facilitation of one group of invasive species by another group (Simberloff and VonHolle 1999). Although the potential for positive interactions is difficult to predict, inclusion of positive interactions is essential to improve understanding of invasion (Richardson et al. 2000a). The invasibility of a site increases with the number of generalist mutualists and the number of other invasive species present.

An example of positive interactions between two invaders that increases species invasive potential is found in the Córdoba Mountains of central Argentina. Invasive plant *Ligstrum* spp. density is four times higher under invasive plant *Pyracantha angustifolia* canopies than under canopies of native plants or in the open (Tecco et al. 2006). Although the invasive *Ligstrum* spp. still occurs on the landscape outside *P. angustifolia* canopy, the presence of *P. angustifolia* increased the invasibility of a site to *Ligstrum* spp. (Tecco et al. 2006). This observation is a clear example of invasional meltdown; the presence of one invasive species increases the invasibility of a site to another invasive species.

### Site environmental conditions

The third set of factors discussed is depicted as the “Site Environmental Condition” side of the invasion triangle and represents environmental or physical conditions of a site that influence its invasibility. These conditions are inherent physical characteristics of a site such as the amount of unused resources and habitat suitability (e.g., temperature and precipitation regimes). Some of the obstacles a new propagule arriving on a site must overcome are environmental conditions such as the need for essential resources (i.e., nutrients, water, space) and conditions conducive for survival and growth. If the environmental conditions are outside the tolerance of a potential invader, it will not establish regardless of how susceptible the resident community or how high the invasive potential of that invader. We discuss two suites of environmental conditions: resources and habitat suitability.

#### Resources

A fundamental site characteristic included in many discussions and hypotheses regarding invasiveness is the presence of unused resources (e.g., light, water, nutrients, space; Elton 1958; Davis et al. 2000; Mack et al. 2000; Shea and Chesson 2002). Essentially, when resource availability is greater than resource uptake by the resident community, the site is thought to be more vulnerable to invasion. Unused resources may be a static property of the system (vacant niche hypothesis, Elton 1958; Mack et al. 2000) where the invader exploits resources that are not utilized by the resident community. Unused resources may also be a dynamic property of the system where a fluctuation or pulse of resource becomes available (variable resource hypothesis, Davis et al. 2000; resource opportunity, Shea and Chesson 2002) and makes the site vulnerable to invasion at a particular point in time or space.

For example, nutrient enrichment was the most influential determinant of invasion by *Dasyhelea* spp. in experimentally created rock pool microcosms (Romanuk and Kolasa 2005). This project manipulated both diversity and nutrients (increasing unused resources), and then monitored natural invasion in outdoor rock pool microcosms. Increased diversity did not confer resistance to invasion, but rather site invasibility was determined by increased resources. Thus, increased resources were the principle mechanism influencing invasibility in the microcosms (Romanuk and Kolasa 2005).

#### Habitat suitability

When investigating site invasibility, many analyses include variables that evaluate the suitability of a site for a particular invader with variables such as temperature (Avery et al. 2010), water availability, length of growing season, soil type, and elevation (e.g., Chambers et al. 2007). In order for a species to establish and become invasive, a level of synchrony between physical characteristics of the native range and the introduced range is required (Alpert et al. 2000). This synchrony is referred to as habitat suitability. Habitat suitability is a function of both climate conditions and physical characteristics of the site. At least some similarity between a species’ home range climate and the climate in the recipient site must exist for invasion to occur (i.e., climate matching, Williamson 1996; Peterson 2003). Physical characteristics of a site that fall into the habitat suitability variable include characteristics such as soil texture, water inundation levels, or temperature regime. Although individual species may respond to physical characteristics and climate in unpredictable ways, a site that has more similar climate and physical characteristics to the native range of an invader may be more susceptible to that invader.

For example, habitat suitability variables best explain the invasibility of *Artemesia tridentata* shrublands by the invader *Bromus tectorum* (Chambers et al. 2007). This project manipulated fire occurrence and biotic resistance, and measured soil nutrients, elevation, soil water, and temperature. Most variation in site invasibility was explained by soil water at low elevations and temperature at high elevations, whereas fire and biotic resistance had only minor effects on site invasibility (Chambers et al. 2007). Habitat suitability variables underlie all species distributions and should be incorporated into any model of invasion.

### External Influences

The final set of factors discussed is depicted as the “External Influence” arrow and represents transient dynamics that are not inherent at a site but still have the ability to modify the risk of invasion. External influences are generated outside the site, impact the ecological integrity of a site, and are often, but not exclusively, anthropogenically generated. External influences act on one or more sides of the triangle, altering the risk of invasion (Fig. 2). An external influence alters the invasibility of a site through alteration of characteristics depicted on one or more sides of the invasion triangle. The underlying concept for external influences is that the native community and environmental characteristics at a site may be a result of current conditions, thus alteration of those conditions may favor invasion. Some examples of invasion ecology hypotheses that examine external influences on invasion risk are changes in land use and disturbance regime, global change, and introduction effort.

#### Changes in land use and disturbance regime

The native community at a site has evolved with a particular land use and disturbance regime, and alteration to that regime has the potential to change invasibility of that site (Hobbs and Huenneke 1992). As human population expands and migrates, land use patterns change from areas with no deliberate modification (wilderness areas) to areas where native ecosystems are exploited (e.g., grazing or logging), intensively managed (agriculture), or urbanized (Hobbs 2000). Even subtle changes in land use have the ability to alter native disturbance regimes (e.g., fire frequency or grazing intensity) and to increase fragmentation and human impacts, all of which could potentially induce a cascade of changes in the community and result in increased invasibility of a site (Alpert et al. 2000). Changes in disturbance frequency or intensity have the potential to severely disrupt ecosystem processes that influences site invasibility. Similarly, changes in natural disturbance regimes may alter community composition and stability, and thus increase unused resources that subsequently increase the risk of invasion (Hobbs and Huenneke 1992). Any site that is subject to a change in land use or disturbance regime is also subject to an alteration of invasibility.

A study of plant invasion in the Mingha Valley of New Zealand found that the invasive species *Anthoxanthum odoratum*, *Holcus lanatus*, *Cerastium fontanum*, and *Hieracium pilosella* were all promoted by anthropogenic disturbances (Jesson et al. 2000). Indicators of anthropogenic disturbances included distance from hiking trails, proximity to backcountry huts, and two flooding regimes in a riparian zone. In this study, disturbance influenced introduction pressure, unused resources, and biotic resistance, all of which combined to increase invasion (Jesson et al. 2000). This result illustrates how anthropogenic alteration of disturbance regime (increased land use and water management) increases site invasibility.

#### Global environmental change

Increasing atmospheric carbon dioxide, alteration of precipitation and temperature regimes, and increasing nitrogen deposition are all examples of global environmental change. Global environmental change is one variable that is likely to affect all potential invasions because changing environments have the potential to modify site conditions so that a new suite of species may become invasive (Hellmann et al. 2008), change the invasive potential of species (Fenn et al. 2003), alter introduction and dispersal patterns (Hellmann et al. 2008; Walther et al. 2009), change current range limits of already established invaders (Hellmann et al. 2008; Walther et al. 2009), and decrease productivity of native species (Zhao and Running 2010). However, responses to global environmental change are likely to be species specific, and thus all nonnative species will not respond similarly. Just like natives, any individual introduced species that benefits from carbon and nitrogen enrichment or responds positively to a change in precipitation and temperature may increase in range or abundance. If global environmental change alters biotic characteristics or environmental conditions of a site, then invasibility of that site will change.

Invasive clam, *Corbicula fluminea*, populations in the Rhine River illustrate the complexity involved when including the influence of climate change in an examination of invasion. An experiment monitored invasive *C. fluminea* performance in ambient and warmed water (+ 3°C) in summer and winter (Weitere et al. 2009). When warming occurred in summer, *C. fluminea* fitness decreased, but when warming occurred in winter, *C. fluminea* fitness increased (Weitere et al. 2009). This result demonstrates the complexity induced by climate change on invasion. The influence of climate change on *C. fluminea* invasion could be positive or negative depending on the season in which warming was more pronounced.

#### Introduction effort

Introduction effort is the number of propagules introduced either once or repeatedly to a site (Lockwood et al. 2005). To reduce confusion, the term “introduction effort” is used in this paper rather than “propagule pressure,” which often includes reproductive output on a site. Attention to introduction effort has increased in relation to invasion (Von Holle and Simberloff 2005; Pysek and Richardson 2006). Movement of humans around the globe has effectively broken down geographic barriers for invasion. The question may not be if a species will be introduced into a new range, but in what quantity will it be introduced. The relationship between introduction effort and invasion is positive: as the number of propagules reaching a site increases, the more likely invasion becomes (Pysek and Richardson 2006). An increase in number of propagules introduced to a new range also could increase the genetic diversity of the invader, which in turn provides more ability for evolution of invasiveness (Lockwood et al. 2005) and provides a buffer against stochastic unfavorable conditions. High introduction effort can also overcome the intrinsic resistance of a site to invasion (Von Holle and Simberloff 2005). Introduction effort is difficult to determine but some sites are prone to higher levels of introduction than others, such as areas with high levels of human recreation or areas in proximity to shipping lanes. Species introduced intentionally (i.e., for landscaping, forage, erosion control) have been chosen specifically for a likelihood of success and therefore may need fewer introductions to become invasive than species introduced accidentally. While not every nonnative species that is introduced in high quantities will become invasive, introduction effort can explain why many invasions seem so idiosyncratic (Lockwood et al. 2005; Colautti et al. 2006); increasing levels of introduction may overcome both site invasibility limitations and low invasiveness of a potential invader.

## Application of the Invasion Triangle

The first step to use the invasion triangle as an organizational framework is to gather information on each set of factors depicted in the invasion triangle (each side and external influences) for the situation in question. The invasion triangle can be generated at any level of data intensity. Information may come from experimental or observational research, literature review, or expert opinion. Although information (especially on new invasions) may be limited, it cannot be overemphasized that the more quantitative the information used in model development, the more reliable and useful the resulting invasion triangle. After information is gathered and organized into an invasion triangle, research needs are easily identified (i.e., if information is only available for two of three sides of the triangle, then research is needed on the third side).

If quantitative data for the invasion triangle is collected in a single study, or enough quantitative information is available for all three sides of the invasion triangle, the next step with the invasion triangle is to scale the sides relative to each other (see “Next Steps” section below for more discussion about scaling sides). The underlying concepts for creating a scaled invasion triangle are: (1) the length of each side reflects the overall risk of invasion due to factors on that side (i.e., the more risk, the longer the side); and (2) the size of the scaled triangle reflects overall invasion (i.e., bigger triangles mean bigger invasion). In the next sections, we discuss how scaled invasion triangles inform quantitative models, communicate information on invasion, and assess potential management actions.

### Informing quantitative models

Sophisticated quantitative models such as invasion risk assessment and invasive species distribution modeling (Guisan and Zimmermann 2000; Mack et al. 2000; Austin 2002; Stohlgren et al. 2010) have recently increased methodologically and in popularity, and are enterprises that will benefit from considering the invasion triangle during model formulation. We propose that formal development of an invasion triangle (i.e., conceptual model formulation) should be the first step in any modeling effort (Guisan and Zimmerman 2000). Development of an invasion triangle requires consideration of the entire set of factors that influences invasion (i.e., invader attributes, site biological characteristics, site environmental conditions, and external influences). Formally developing an invasion triangle ensures that as much information (e.g., ecological knowledge, Austin 2002) as possible for a given invasion situation is utilized and assures that data completeness is appraised, thus improving the resulting quantitative models (Austin 2002). Utilization of the invasion triangle as the underlying organizational step provides a consistency in communication and conceptual framework among studies. An increase in consistency among studies of invasion could help invasion biology move past debates about single hypothesis and leads to a better understanding of species invasion. Finally, the invasion triangle has no inherent spatial scale and thus can be developed for models at any scale determined to be appropriate.

### Communication

The graphical nature of the invasion triangle easily communicates information on any given invasion. Scaled invasion triangles are depicted with sides scaled relative to each other and with the length of each side reflecting the site invasibility or species invasion potential (i.e., the greater invasive potential an invader has in a particular site, the longer that side should be depicted). Both the size and shape of the resulting invasion triangle conveys information about invasion (Fig. 2, left side): longer sides have more impact on invasion than shorter sides, and larger triangles convey more risk of invasion than smaller triangles. After an initial invasion triangle is developed, external influences may be incorporated, if needed, and alternate triangles developed (Fig. 2, right side). It is worth noting that if the characteristics represented on a given side of the invasion triangle are determined to be resistant to invasion (e.g., cold and infertile environmental conditions in an invasion triangle for a frost-intolerant nitrophilic potential invader), then the value, and thus side length, produced would be approximately zero; thus, that particular invasion in that particular site would not be expected to occur.

### Assessing management actions

Increasingly, management actions are undertaken to combat invasion. Management is an enterprise that will benefit from the invasion triangle because developing an invasion triangle ensures that as much information is considered as possible. The graphic depiction of the invasion situation in question helps evaluate potential management applications. Management actions, like external influences, can affect one or more sides of the invasion triangle. Management actions often have limited success (Parks and Panetta 2009), perhaps because they frequently target conditions depicted on only one side of the invasion triangle. Examples of these actions include: direct removal of the invaders, reintroduction of natives, introduction of biocontrol agents, and alteration of resources (Grice 2009). For invasion to be significantly affected when management actions only target conditions depicted on one side of the invasion triangle, either (1) that specific condition must be the dominant influence; or (2) the influence of that specific condition must be reduced to near zero.

Occasionally management actions inadvertently increase invasion (Parks and Panetta 2009). Consideration of how a management action may influence each side of the invasion triangle could help anticipate these undesirable outcomes. For a hypothetical example, a management action that mechanically removes terrestrial plant invaders may also remove native plants and increase unused resources. Thus, although the invader attribute side decreases (due to decreased population numbers), the site biotic and site environmental sides become more conducive to invasion resulting in increased invasibility. An alternate management decision such as a more precise targeted application to remove invaders, but not affect the natives, would result in much less unused resources.

Blumenthal et al. (2005) provide an example of a restoration project that addressed factors on each side of the invasion triangle. This study evaluated the effect of several different restoration treatments on plant invasion. The treatments included: increased biotic resistance (added native seed), increased resources (fertilization), and increased introduction pressure (added invader seeds). Invasion was lowest in sites with increased biotic resistance (added native seed), higher in sites with the most unused resources (fertilized), and higher in sites with increased introduction pressure (Blumenthal et al. 2005). From these results, we infer that a management treatment that targeted three aspects of the invasion triangle, resulting in increased biotic resistance (site biotic characteristic side), decreased resources (site environmental condition side), and decreased introduction pressure (external influence), would best manage invasion at this site.

## Case Studies

### Hieracium lepidulum conceptual invasion triangle from literature review

**Figure 3:**
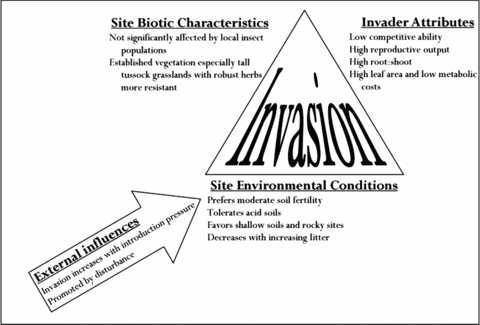
Conceptual invasion triangle for *Heracium lepidulum* in New Zealand based on information gathered in a literature review of Meffin et al. 2010; Radford et al. 2006, 2007, 2009, 2010; Rose and Frampton 1999; Syrett and Smith 1998; and Wiser et al. 1998. Note that this triangle is conceptual and that sides of the triangle have not been scaled.

*Hieracium lepidulum* is an aster originally found in Europe that now has an invaded range that includes subalpine areas in New Zealand (Radford et al. 2007). Invaded areas incur a decline in native species and decreased habitat quality (Radford et al. 2007). Information on factors depicted on each side of the invasion triangle can be gathered from a literature review and used to generate a conceptual invasion triangle (Fig. 3). Invader attributes of *H. lepidulum* include high reproductive output (Rose and Framton 1999), high root:shoot (Radford 2006), high leaf area with low metabolic costs (Radford et al. 2009), and low competitive ability (Radford et al. 2006, 2007, 2010; Meffin et al. 2010). Investigations into site biotic characteristics that influence invasibility reveal that local insect populations in invaded areas do not significantly affect *H. lepidulum* (Syrett and Smith 1998) and that sites with established vegetation, especially tall tussock grasslands with a robust herbaceous component, experience less invasion than other sites (Wiser et al. 1998; Rose and Frampton 1999). Studies on site environmental conditions in relation to *H. lepidulum* invasion determined that *H. lepidulum* performs optimally with moderate soil fertility, tolerates acid soils, and prefers shallow soils and rocky sites (Wiser et al. 1998; Radford et al. 2006, 2007, 2010), and that *H. lepidulum* density decreases with increasing litter cover (Rose and Framton 1999). Both high introduction pressure (Rose and Frampton 1999; Meffin et al. 2010) and increased disturbance (Wiser et al. 1998; Rose and Frampton 1999; Radford et al. 2007, 2010) are external influences that increase the invasibility of a site to *H. lepidulum*.

Based on the invasion triangle for *H. lepidulum* (Fig. 3), we can evaluate potential management actions. Considering the invader attributes side of the triangle, we anticipate that an action that reduced invader biomass would be ineffective in reducing invasion due to the low metabolic costs in leaves. However, any action that decreased *H. lepidulum*'s normally high reproductive output has potential to reduce invasion. Based on the site biological characteristics side of the triangle, we do not expect *H. lepidulum* to be affected by insect “enemies.” However, if a management action increases plant diversity and promotes tall tussock grassland vegetation, invasibility might be decreased by biotic resistance. Based on the site environmental condition side of the triangle, a management action that increased litter cover would be expected to decrease invasibility. A management action that addressed external influences and decreased disturbance and introduction pressure also decreases invasibility. As discussed above, management actions that reduce invasibility by acting on more than one aspect of the triangle should decrease invasibility more than an action that just acts on one side. Thus, a management action that addresses more than one set of factors depicted in the invasion triangle, for example, reduced grazing and limited disturbance (external influences); reduced soil fertility and increased litter (site environmental conditions); and promotes native vegetation (site biotic characteristics) might be the preferred action based on this invasion triangle.

### Alliaria petiolata, Berberis thunbergii, and Microstegium vimineum scaled invasion triangles

**Figure 4:**
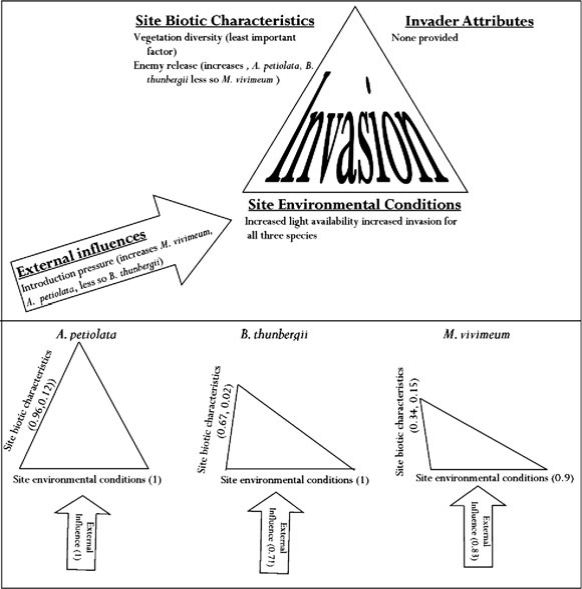
Invasion triangles for *Alliaria petiolata*, *Berberis thunbergii*, and *Microstegium vimineum* based on information provided by Eschtruth and Battles (2009). Top panel is the conceptual invasion triangle, and the bottom panel is triangles for each species with the sides scaled relative to each other. The values in the parentheses reported relative importance (Akaike Information Criteria weights) of each variable. The values on the site biotic side are first enemy release and second are species diversity. No variables were reported that corresponded with the invader attribute side of the triangle, thus as a default, that side is scaled to one although other values may also be appropriate.

Invasions by *Alliaria petiolata*, *Berberis thunbergii*, and *Microstegium vimineum* were studied in the Delaware Water Gap National Recreation Area in northeastern United States (Eschtruth and Battles 2009). This study evaluated several factors that are integrated easily into the invasion triangle: site biotic characteristics of vegetation diversity and enemy release; site environmental condition of light availability; and external influence of introduction pressure (Fig. 4, top panel). Eschtruth and Battles (2009) report that the most important variables affecting *A. petiolata* invasion are site environmental conditions and enemy release, whereas species diversity is much less important. Increasing light is also the most important variable affecting invasions of *B. thunbergii* and *M. vimineum*, whereas enemy release has much less importance, and species diversity has almost no importance (Eschtruth and Battles 2009). We constructed scaled invasion triangles individually for each species using the relative importance of the variables provided from this study (Fig. 4, bottom panel). The sides of each triangle are scaled relative with each other. However, because no variable that corresponds to the invader attribute side of the triangle was provided, that side is scaled to a default value of one, although other values may also be appropriate. The invasion triangle for *A. petiolata* is the largest and the triangle for *M. vimimeum* is the smallest (Fig. 4, bottom panel). This ranking of scaled invasion triangles corresponds to the reported increase in percent cover (*A. petiolata* 12.5%, *B. thunbergi* 6.4%, and *M. vimineum* 3.1%, Eschtruth and Battles 2009) for these species in this area over the duration of this study.

### Invasion triangle generation from qualitative information

**Figure 5:**
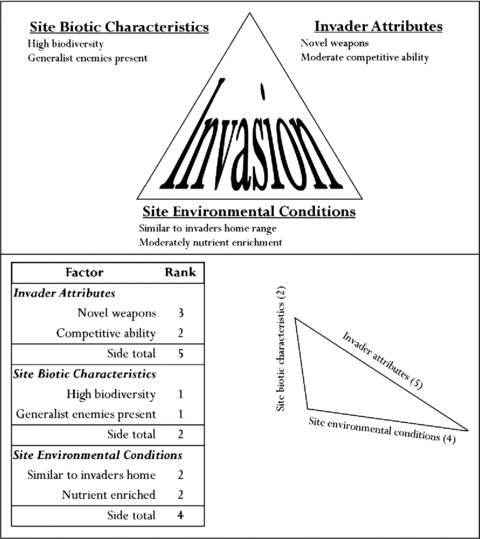
Qualitative invasion triangle. Factors are ranked based on expert opinion, familiarity with the situation, or information from similar situations. Top panel is the conceptual invasion triangle and the bottom panel is the scaled invasion triangle. Sides are scaled relative to each other.

In many invasion situations, quantitative data may not be available; however, an invasion triangle can be generated using expert opinion, familiarity with the situation in question, and results from similar situations. Applicable factors may be ranked in order of relative contribution to risk of species invasion and given values based on those ranks (i.e., high = 3, moderate = 2, low = 1, none = 0, although a larger range of values may also be appropriate). Thus factors that result in high resistance to invasion (such as biodiversity or limited resources) should get low ranks. For example, a potential invader that possesses novel weapons and has moderate competitive ability, both factors that contribute to the risk of invasion, may be ranked at 5 (novel weapons at a rank of 3 plus competitive ability at a rank of 2). The biotic conditions at a potentially invaded site that has high biodiversity and many generalist enemies, which both contribute to invasion resistance, might be given a rank of 2 (1 for each biodiversity and enemies). Finally, if the potentially invaded site environmental conditions were similar to the environmental conditions of the potential invaders’ home range and the site had moderate nutrient enrichment, then a rank of 4 may be given (2 for habitat suitability and 2 for nutrient enrichment). With this information, both a conceptual (Fig. 5, top panel) and a scaled (Fig. 5, bottom panel) triangle can be developed. In turn, these triangles can be used to communicate and to generate research and management questions and priorities.

## Next Steps

The invasion triangle has been presented as a conceptual framework useful for many enterprises such as, but not limited to, focusing research, facilitating communication, and directing management. The required steps to move the invasion triangle from strictly a conceptual framework to a quantitative method to evaluate invasion are to (1) develop techniques to appropriately scale the sides relative to each other (especially when the number of factors on the sides is different); and (2) determine if scaled triangle size is proportional to actual invasion.

In many invasion situations, much more information is available for the factors depicted on one side of the triangle than other sides, and information on the effects of external factors on each side may be limited. This inequality can be a challenge for quantitative scaling of the sides relative to each other (i.e., invasion triangles cannot be compared if one side of a triangle is longer simply because more information is available on that side). A method to overcome this challenge would be to use experiments or observational studies combined with a statistical approach such as information-theoretic model averaging approaches and AIC (Akaike information criterion) weights (e.g., Eschtruth and Battles 2009) that provide a measure of importance of either individual variables or groups of variables relative to each other (Burnham and Anderson 2002; Grueber et al. 2011).

Another step is a quantitative evaluation of the predictive ability of the invasion triangle. Quantitative data need to be collected for factors depicted on each side of the triangle, appropriately scaled, and then these measures should be used to calculate invasion triangle size. These data can be collected either for one species at a number of different sites (an extension of the first example case study above) or for a number of species in one locale (analogous to the second example case study). The relationship between triangle size and invasion ideally should be directly proportional. Appropriate hypotheses to test the quantitative aspects of the invasion triangle include: (1) larger triangles reflect more risk of invasion; (2) if factors depicted on one side increase/decrease, invasion risk will increase/decrease; and (3) if factors depicted on one side are manipulated to be resistant to invasion (a value near zero), invasion should not occur. We note that the appropriate measure of invasion triangle size is area, not perimeter. Intuitively (and mathematically) for triangles with a constant perimeter, area approaches zero as the length of any one side also approaches zero. Potential hypotheses to test the appropriateness of triangle area include: (1) different combinations of side lengths that give similar area result in similar invasion; and (2) a percentage change in one side always results in a smaller percentage change in invasion (i.e., a 50% change in one side would not result in a 50% change in invasion due to the influence of other sides).

Results from the second case study, where sides of the triangle were scaled relative to each other and size of the invasion triangle was concordant with invasion, indicate a likelihood that scaled invasion triangles have potential to predict invasion. With adoption of the invasion triangle as a conceptual framework, we believe more data will become available to examine the quantitative dimension of the invasion triangle. Nonetheless, with or without any quantitative development, the invasion triangle has utility and benefit as a conceptual framework.

## Conclusion

We have introduced a conceptual framework for species invasion based on a simple triangle. The three sides of the invasion triangle encapsulate the major suites of processes that influence invasion: attributes of the invader, biotic characteristics of the potentially invaded site, and environmental conditions of the potentially invaded site. Also included in the invasion triangle are external influences such as climate change and alteration of land use that independently modify invasion processes. We propose that the organizational step of creating an invasion triangle benefits research, communication, quantitative modeling, and management in regard to species invasion. Its intuitive, straightforward structure with flexible data requirements makes the invasive triangle easy to apply in both research and management contexts.

## References

[b1] Alpert P, Bone E, Holzapfel C (2000). Invasiveness, invasibility and the role of environmental stress in the spread of non-native plants. Perspect. Plant Ecol.

[b2] Austin MP (2002). Spatial prediction of species distribution: an interface between ecological theory and statistical modeling. Ecol. Model.

[b3] Avery ML, Engeman RM, Keacher KL, Humphrey JS, Bruce WE, Mathies TC, Mauldin RE (2010). Cold weather and the potential range of invasive Burmese pythons. Biol. Invasions.

[b4] Baker H (1974). The evolution of weeds. Annu. Rev. Ecol. Syst.

[b5] Barney JN, Whitlow TH (2008). A unifying framework for biological invasions: the state factor model. Biol. Invasions.

[b6] Blossey B, Notzold R (1995). Evolution of increased competitive ability in invasive nonindigenous plants—a hypothesis. J. Ecol.

[b7] Blumenthal DM, Jordan RJ, Svenson EL (2005). Effect of prairie restoration on weed invasions. Agr. Ecosyst. Environ.

[b8] Bossdorf O, Auge H, Lafuma L, Rogers WE, Siemann E, Prati D (2005). Phenotypic and genetic differentiation between native and introduced plant populations. Oecologia.

[b9] Burnham K, Anderson D (2002). Model selection and multimodel inference: a practical information-theoretic approach.

[b10] Buswell JM, Moles AT, Hartley S (2011). Is rapid evolution common in introduced plant species?. J. Ecol.

[b11] Callaway RM, Aschehoug ET (2000). Invasive plants versus their new and old neighbors: a mechanism for exotic invasion. Science.

[b12] Catford JA, Jansson R, Nilsson C (2009). Reducing redundancy in invasion ecology by integrating hypotheses into a single theoretical framework. Divers. Distribut.

[b13] Chambers JC, Roundy BA, Blank RR, Meyer SE, Whittaker A (2007). What makes Great Basin sagebrush ecosystems invasible by *Bromus tectorum*. Ecol. Monogr.

[b14] Clements DR, Ditommaso A (2011). Climate change and weed adaptation: can evolution of invasive plants lead to greater range expansion than forecasted?. Weed Res.

[b15] Colautti RI, Grigorovich IA, MacIsaac HJ (2006). Propagule pressure: a null model for biological invasions. Biol. Invasions.

[b16] Cuddington K, Hastings A (2004). Invasive engineers. Ecol. Model.

[b17] Cuddington K, Wilson WG, Hastings A (2009). Ecosystem engineers: feedback and population dynamics. Am. Nat.

[b18] Davis MA, Grime JP, Thompson K (2000). Fluctuating resources in plant communities: a general theory of invasibility. J. Ecol.

[b19] Davis MA, Pergl J, Truscott A, Kollmann J, Bakker J, Domenech R, Prach K, Richard A, Veeneklaas R, Pysek P (2005). Vegetation change: a reunifying concept in plant ecology. Perspect. Plant Ecol.

[b20] Elton C (1958). The ecology of invasions by animals and plants.

[b21] Eschtruth AK, Battles JJ (2009). Assessing the relative importance of disturbance herbivory, diversity, and propagule pressure in exotic plant invasion. Ecol. Monogr.

[b22] Facon B, Genton BJ, Shykoff J, Jarne P, Estoup A, David P (2006). A general eco-evolutionary framework for understanding bioinvasions. Trends Ecol. Evol.

[b23] Farrer EC, Goldberg DE (2009). Litter drives ecosystem and plant community changes in cattail invasion. Ecol. Appl.

[b24] Fenn ME, Baron JS, Allen EB, Rueth HM, Nydick KR, Geiser L, Bowman WD, Sickman JO, Meixner T, Johnson DW (2003). Ecological effects of nitrogen deposition in the western United States. Bioscience.

[b25] Foxcroft LC, Pickett STA, Cadenasso ML (2011). Expanding the conceptual frameworks of plant invasion ecology. Perspect. Plant Ecol.

[b26] Goldberg DE, Landa K (1991). Competitive effect and response—hierarchies and correlated traits in the early stages of competition. J. Ecol.

[b27] Grice T, Clout M, Williams P (2009). Invasive species management: a handbook of principles and techniques.

[b28] Grueber CE, Nakagawa S, Laws RJ, Jamieson IG (2011). Multimodel inference in ecology and evolution: challenges and solutions. J. Evol. Biol.

[b29] Guisan A, Zimmermann NE (2000). Predictive habitat distribution models in ecology. Ecol. Model.

[b30] Gurevitch J, Fox GA, Wardle GM, Inderjit, Taub D (2011). Emergent insights from the synthesis of conceptual frameworks for biological invasions. Ecol. Lett.

[b31] Harris DJ, Smith KG, Hanly PJ (2011). Occupancy is nine-tenths of the law: occupancy rates determine the homogenizing and differentiating effects of exotic species. Am. Nat.

[b32] Haubensak KA, D'Antonio CM (2011). The importance of nitrogen-fixation for an invader of a coastal California grassland. Biol. Invasions.

[b33] Hellmann JJ, Byers JE, Bierwagen BG, Dukes JS (2008). Five potential consequences of climate change for invasive species. Conserv. Biol.

[b34] Hierro JL, Maron JL, Callaway RM (2005). A biogeographical approach to plant invasions: the importance of studying exotics in their introduced and native range. J. Ecol.

[b35] Hobbs R, Mooney H, Hobbs R (2000). Invasive species in a changing world. Land-use changes and invasions.

[b36] Hobbs RJ, Huenneke LF (1992). Disturbance, diversity, and invasion—implications for conservations. Conserv. Biol.

[b37] Jesson L, Kelly D, Sparrow A (2000). The importance of dispersal, disturbance, and competition for exotic plant invasions in Arthur's Pass National Park, New Zealand. New Zeal. J. Bot.

[b38] Jones CG, Lawton JH, Shachak M (1994). Organisms as ecosystem engineers. Oikos.

[b39] Keane RM, Crawley MJ (2002). Exotic plant invasions and the enemy release hypothesis. Trends Ecol. Evol.

[b40] Kennedy TA, Naeem S, Howe KM, Knops JMH, Tilman D, Reich P (2002). Biodiversity as a barrier to ecological invasion. Nature.

[b41] Lambrinos JG (2004). How interactions between ecology and evolution influence contemporary invasion dynamics. Ecology.

[b42] Levine JM (2000). Species diversity and biological invasions: relating local process to community pattern. Science.

[b43] Levine JM, Adler PB, Yelenik SG (2004). A meta-analysis of biotic resistance to exotic plant invasions. Ecol. Lett.

[b44] Lockwood JL, Cassey P, Blackburn T (2005). The role of propagule pressure in explaining species invasions. Trends Ecol. Evol.

[b45] Lonsdale WM (1999). Global patterns of plant invasions and the concept of invasibility. Ecology.

[b46] Mack RN, Simberloff D, Lonsdale WM, Evans H, Clout M, Bazzaz FA (2000). Biotic invasions: causes, epidemiology, global consequences, and control. Ecol. Appl.

[b47] Maron JL, Vila M (2001). When do herbivores affect plant invasion? Evidence for the natural enemies and biotic resistance hypotheses. Oikos.

[b48] Meffin R, Miller AL, Hulme PE, Duncan RP (2010). Experimental introduction of the alien plant *Hieracium lepidulum* reveals no significant impact on montane plant communities in New Zealand. Divers. Distribut.

[b49] Mitchell CE, Agrawal AA, Bever JD, Gilbert GS, Hufbauer RA, Klironomos JN, Maron JL, Morris WF, Parker IM, Power AG (2006). Biotic interactions and plant invasions. Ecol. Lett.

[b50] Parker IM, Gilbert GS (2004). The evolutionary ecology of novel plant-pathogen interactions. Annu. Rev. Ecol. Evol. Syst.

[b51] Parks J, Pannetta F, Clout M, Williams P (2009). Eradication of invasive species: progress and emerging issues in the 21st century, nvasive species management: a handbook of principles and techniques.

[b52] Peterson AT (2003). Predicting the geography of species’ invasions via ecological niche modeling. Q. Rev. Biol.

[b53] Pysek P, Richardson DM (2006). The biogeography of naturalization in alien plants. J. Biogeogr.

[b54] Radford IJ, Dickinson KJM, Lord JM (2006). Nutrient stress and performance of invasive *Hieracium lepidulum* and co-occurring species in New Zealand. Basic Appl. Ecol.

[b55] Radford IJ, Dickinson KJM, Lord JM (2007). Functional and performance comparisons of invasive *Hieracium lepidulum* and co-occurring species in New Zealand. Austral Ecol.

[b56] Radford IJ, Dickinson KJM, Lord JM (2009). Does the invader *Hieracium lepidulum* have a comparative growth advantage over co-occurring plants? High leaf area and low metabolic costs as invasive traits. New Zeal. J. Bot.

[b57] Radford IJ, Dickinson KJM, Lord JM (2010). Does disturbance, competition or resource limitation underlie *Hieracium lepidulum* invasion in New Zealand? Mechanisms of establishment and persistence, and functional differentiation among invasive and native species. Austral Ecol.

[b58] Richardson DM, Allsopp N, D'Antonio CM, Milton SJ, Rejmanek M (2000a). Plant invasions—the role of mutualisms. Biol. Rev.

[b59] Richardson DM, Pysek P, Rejmanek M, Barbour MG, Panetta FD, West CJ (2000b). Naturalization and invasion of alien plants: concepts and definitions. Divers. Distribut.

[b60] Romanuk TN, Kolasa J (2005). Resource limitation, biodiversity, and competitive effects interact to determine the invasibility of rock pool microcosms. Biol. Invasions.

[b61] Rose AB, Frampton CM (1999). Effects of microsite characteristics on Hieracium seedling establishment in tall- and short-tussock grasslands, Marlborough, New Zealand. New Zeal. J. Bot.

[b62] Sakai AK, Allendorf FW, Holt JS, Lodge DM, Molofsky J, With KA, Baughman S, Cabin RJ, Cohen JE, Ellstrand NC (2001). The population biology of invasive species. Annu. Rev. Ecol. Syst.

[b63] Schierenbeck KA, Ellstrand NC (2009). Hybridization and the evolution of invasiveness in plants and other organisms. Biol. Invasions.

[b64] Shea K, Chesson P (2002). Community ecology theory as a framework for biological invasions. Trends Ecol. Evol.

[b65] Simberloff D, Von Holle B (1999). Positive interactions of nonindigenous species: invasional meltdown?. Biol. Invasions.

[b66] Stevens RB, Horsfall JG, Dimond AE (1960). Plant pathology, an advanced treatise.

[b67] Stohlgren TJ, Barnett DT, Kartesz J (2003). The rich get richer: patterns of plant invasions in the United States. Front. Ecol. Environ.

[b68] Stohlgren TJ, Ma P, Kumar S, Rocca M, Morisette JT, Jarnevich CS, Benson N (2010). Ensemble Habitat Mapping of Invasive Plant Species. Risk Analysis.

[b69] Suttle KB, Hoddle MS (2006). Engineering enemy-free space: an invasive pest that kills its predators. Biol. Invasions.

[b70] Syrett P, Smith LA (1998). The insect fauna of four weedy Hieracium (Asteraceae) species in New Zealand. New Zeal. J. Zool.

[b71] Tecco PA, Gurvich DE, Diaz S, Perez-Harguindeguy NP, Cabido M (2006). Positive interaction between invasive plants: the influence of *Pyracantha angustifolia* on the recruitment of native and exotic woody species. Austral Ecol.

[b72] Valery L, Fritz H, Lefeuvre JC, Simberloff D (2008). In search of a real definition of the biological invasion phenomenon itself. Biol. Invasions.

[b73] Vila M, Weiner J (2004). Are invasive plant species better competitors than native plant species? Evidence from pair-wise experiments. Oikos.

[b74] Von Holle B, Simberloff D (2005). Ecological resistance to biological invasion overwhelmed by propagule pressure. Ecology.

[b75] Walther G-R, Roques A, Hulme PE, Sykes MT, Pysek P, Kühn I, Zobel M, Bacher S, Botta-Dukát Z, Bugmann H (2009). Alien species in a warmer world: risks and opportunities. Trends Ecol. Evol.

[b76] Wardle DA (2001). Experimental demonstration that plant diversity reduces invasibility—evidence of a biological mechanism or a consequence of sampling effect?. Oikos.

[b77] Weitere M, Vohmann A, Schulz N, Linn C, Dietrich D, Arndt H (2009). Linking environmental warming to the fitness of the invasive clam *Corbicula fluminea*. Glob. Change Biol.

[b78] Williamson M (1996). Biological invasions.

[b79] Wiser SK, Allen RB, Clinton PW, Platt KH (1998). Community structure and forest invasion by an exotic herb over 23 years. Ecology.

[b80] Zhao MS, Running SW (2010). Drought-induced reduction in global terrestrial net primary production from 2000 through 2009. Science.

